# Leprosy among new child cases in China: Epidemiological and clinical analysis from 2011 to 2020

**DOI:** 10.1371/journal.pntd.0011092

**Published:** 2023-02-17

**Authors:** Jiayi Peng, Peiwen Sun, Le Wang, Hongsheng Wang, Siyu Long, Mei-Wen Yu

**Affiliations:** 1 National Center for Leprosy Control, Chinese Center for Disease Control and Prevention, Hospital for Skin Diseases (Institute of Dermatology), Chinese Academy of Medical Sciences and Peking Union Medical College, Nanjing, Jiangsu, China; 2 Beijing Chao-Yang Hospital, Capital Medical University, National Clinical Research Center for Skin and Immune Diseases, Beijing, China; Johns Hopkins University, UNITED STATES

## Abstract

**Background:**

Leprosy, caused by *Mycobacterium leprae* infection, mainly affects skin and peripheral nerves and may further lead to disability and deformity if not treated timely. The new case detection rate of leprosy in children reflects the active transmission of leprosy infection. This study aims to present the epidemiology and clinical characteristics of new leprosy cases in children in China from 2011 to 2020.

**Methodology/Principal findings:**

All data from leprosy patients younger than 15 years old were extracted from the Leprosy Management Information System in China (LEPMIS). Statistical Package for the Social Sciences (SPSS) version 12.0 was used for descriptive and analytical statistics of the epidemiological and clinical indicators by the Mann-Whitney test, Kruskal-Wallis test, and Fisher’s exact test. And geographical distribution was analyzed by ArcGIS 10.5. A total of 152 pediatric new cases of leprosy were found over the last decade. The new case detection rate of pediatric leprosy cases decreased from 0.13 to 0.02 per 1,000,000 population over the last ten years. New pediatric cases had a higher new case detection rate in Guizhou, Sichuan, and Yunnan Provinces. All but 7 provinces in China achieved zero new child case for consecutive five years. The onset of leprosy peaked between 10 and 14 years of age, and the male to female ratio was 1.71:1. Pediatric patients were predominantly infected from symptomatic household adult contacts HHCs. Multibacillary leprosy (MB) was the most common. However, a low proportion of patients developed leprosy reaction and grade 2 disability.

**Conclusions/Significance:**

The new case detection rate of pediatric leprosy cases has decreased over the past ten years in China. Spatial analysis indicated clusters in high-endemic areas. Leprosy transmission has stopped in the majority of provinces in China. However, sporadic cases may continue to exist for a long time. Active surveillance especially contact tracing should be focused on in future plan for management of leprosy, and interventions in leprosy clusters should be prioritized.

## Author summary

Leprosy, also known as Hansen’s disease, has long been a great concern in developing countries. Leprosy imposes a substantial burden on patients and society. This disease can impair the skin, nerves, feet, and legs, and then progress to causing deformities, social stigma, and discrimination. The new case detection rate of pediatric leprosy is an essential indicator reflecting recent transmission of the infection. To better understand the epidemiology and clinical characteristics of leprosy cases among children in China, we collected data on new pediatric leprosy cases over the past 10 years from LEPMIS and performed an analysis with different classification groups. We found 152 new pediatric leprosy cases, and the new case detection rate decreased from 0.13 in 2011 to 0.02 in 2020 per 1,000,000 population. Leprosy was more frequent in males, those 10 to 14 years old, HHCs, and MB. The new cases were concentrated in highly endemic areas. The data indicate that active transmission of leprosy infection will still be ongoing in high-endemic areas. Active surveillance, especially contact tracing, plays an important role in interrupting the infection caused by *Mycobacterium leprae* to pursue the elimination of leprosy.

## Introduction

Leprosy is a chronic communicable disease caused by *Mycobacterium leprae*. If not diagnosed or treated timely, patients with leprosy might suffer severe disability and deformity, which places burdens on their families and on society as a whole [1,2].

With the introduction of multidrug therapy (MDT), the prevalence rate of leprosy cases in China has declined continuously over the past decades, reaching the elimination standard of 1/10000 set by the WHO [3–6]. However, new leprosy cases have occurred in recent years. The trend of pediatric new case detection rate reflects the recent transmission of leprosy infection in the community [7]. From 1990 to 1998, 19453 new leprosy cases were detected in China, with 781 cases occurring among children and accounting for 4% [8]. From 2001 to 2010, pediatric leprosy new cases fell slightly, reaching 78 between 2009 and 2010 [9]. There were 106 new leprosy cases among children under 15 years old from 2011 to 2015, accounting for 4.4% of all new leprosy cases in China [10]. The new case detection rate among children declined globally from 2014 to 2018 [11,12]. However, the new case detection rate of new pediatric leprosy cases and its temporal trends in China over the last ten years remain unknown.

Socio-epidemiological and clinical characteristics are important indicators that illustrate the route of transmission and the effectiveness of prevention and control strategies. New pediatric leprosy cases tend to occur in children between the ages of 10 to 14. Previous surveys reflected that the majority of pediatric leprosy new cases were paucibacillary leprosy (PB), while other studies found that MB was the most common [13–15]. Operational classification reflects disease severity [7]. The HHCs of index patients have been confirmed to be associated with a higher risk of developing leprosy [14,16]. Meanwhile, household contacts account for the majority of pediatric leprosy new cases [14,15]. To better achieve the target of zero leprosy infections and zero new pediatric cases with grade 2 disability (G2D) set by the WHO, it is urgent to evaluate the trend and features of new pediatric leprosy cases over the last decade. To better understand the transmission pattern of infection caused by *M*. *leprae* and provide basic support for leprosy control and prevention strategies, this article analyzed socio-epidemiological and clinical characteristics and further described temporal trends and spatial distribution.

## Methods

### Ethics statement

Approval for conducting this epidemiological study was received from the Institute of Dermatology, Chinese Academy of Medical Sciences & Peking Union Medical College. Only national or provincial leprosy physicians had access to extract the data from LEPMIS, and the personal information of leprosy patients was well protected. Since personal information was all anonymous, formal informed consent was not required.

### Data sources and indicator definitions

All epidemiological and clinical data of pediatric leprosy new cases from 2011 to 2020 were collected from LEPMIS. The annual population of children was collected from the National Bureau of Statistics. LEPMIS was first put into use in 2010 and has been updated to the 2.0 version. There are 34 provincial-level administrative regions in China. All data on leprosy cases in Chinese mainland were collected. According to the incidence and prevalence of leprosy and the national leprosy-control plan (2011–2020), provinces were divided into high-endemic areas (Jiangxi, Hunan, Guangxi, Hainan, Sichuan, Chongqing, Guizhou, Yunnan, Tibet), medium-endemic areas (Jiangsu, Zhejiang, Anhui, Fujian, Shandong, Hubei, Guangdong, Shaanxi, Gansu, Xinjiang), and low-endemic areas (Beijing, Tianjin, Hebei, Shanxi, Mongolia, Liaoning, Jilin, Heilongjiang, Shanghai, Henan, Qinghai, Ningxia).

Epidemiological and clinical indicators collected by LEPMIS included sex, age at diagnosis, geographical distribution, diagnostic delay period, contact type, detection mode, number of lesions and affected nerves, grade of disability, skin slit-smear positivity, and leprosy reaction. Household contacts (HHCs) were defined as individuals who have resided in the household and shared meals with the patient for at least 6 months. The annual pediatric new case detection rate was calculated as the annual pediatric leprosy new cases divided by the number of children every year. The diagnostic delay period was defined as the period from the occurrence of symptoms to the diagnosis of leprosy. In terms of age, leprosy cases could be divided into three groups (0 to 4, 5 to 9, and 10 to 14). MB is defined as a patient of leprosy with more than 5 skin lesions or involved with nerve damage or with positive slit-skin smear, regardless of the number of skin lesions while PB is defined as a patient of leprosy with 1 to 5 skin lesions without occurrence of bacilli in a slit-skin smear [17]. According to Ridley and Jopling’s classification, clinical forms of leprosy were classified as indeterminate (I), tuberculoid tuberculoid (TT), borderline tuberculoid (BT), mid-borderline (BB), borderline lepromatous (BL), or lepromatous lepromatous (LL) [18]. Leprosy reactions consist of Type I reaction and Type II reaction. The degree of disability was simplified as grades 0, 1, and 2 according to the WHO disability classification [19]. The disability degree of pediatric patients was evaluated by dermatologists with extensive knowledge and clinical experience on leprosy.

### Statistical analysis

All data were stored and processed in Excel 2007. Numerical variables are displayed as the mean ± standard deviation (SD), and categorical variables are presented as frequencies and percentages. The socio-epidemiological data and clinical information are displayed in divided groups according to WHO classification. Mann–Whitney test and Kruskal-Wallis test was used to analyze numerical variables. Fisher’s exact test was applied for categorical variables. All analyses were carried out in SPSS version 12.0. A *P* value < 0.05 was considered to be statistically significant. The spatial distribution of pediatric new leprosy cases in Yunnan, Guizhou and Sichuan was analyzed by ArcGIS software version 10.5 (Environmental Systems Research Institute, Inc, Redlands, CA, USA).

## Results

### Overall trend

**Fig 1:**
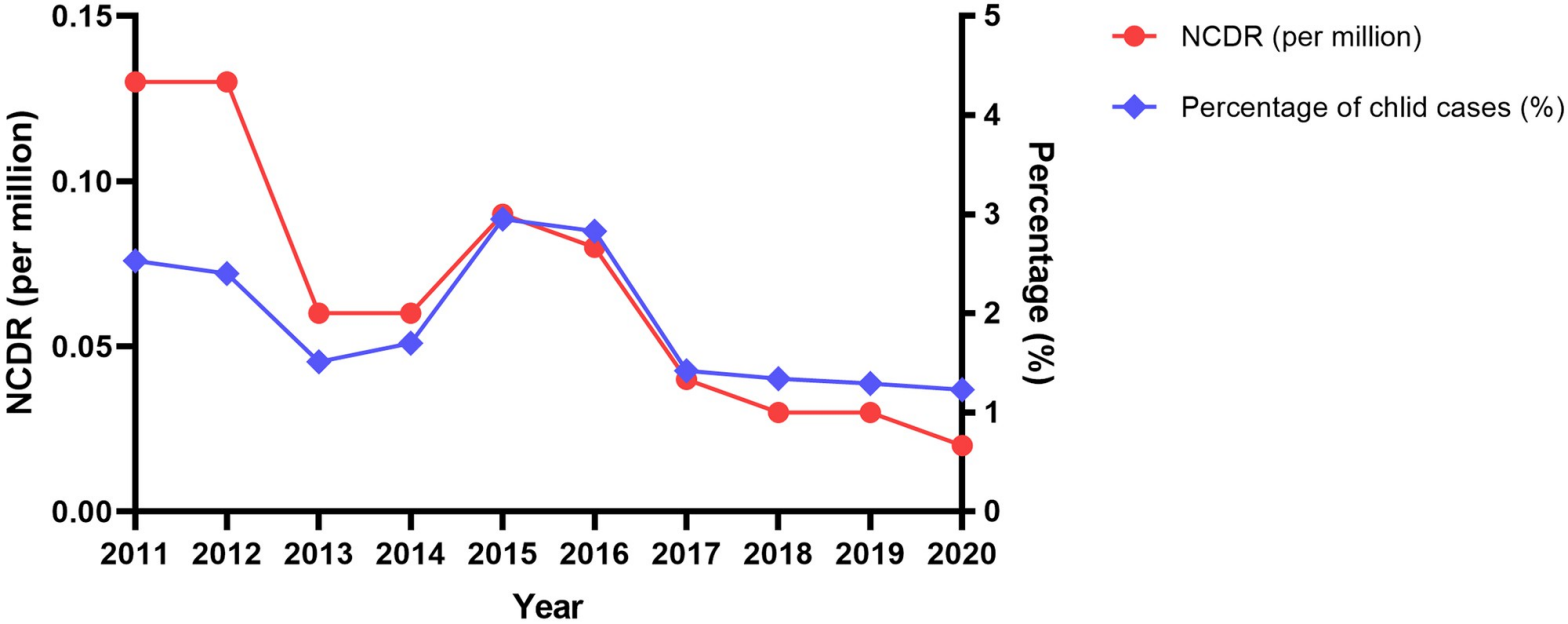
Temporal trend of pediatric leprosy new case detection rates and the proportion of children in new leprosy cases in China, 2011–2020.

From 2011 to 2020, we found 152 new leprosy cases in children in China, with a new case detection rate of 0.60 per million population. Over the past ten years, the new case detection rate of pediatric leprosy new cases in 2011 was the highest (0.13 per million population), while the new case detection rate in 2020 was the lowest (0.02 per million population) (Fig 1). Despite a mild increasing tendency from 2014 to 2015, the new case detection rate of pediatric leprosy decreased dramatically over the last ten years.

### Epidemiological characteristics

The distribution of pediatric leprosy new cases differed in 31 provinces. No new child cases with leprosy were reported in 16 provinces. From 2011 to 2020, provinces with more than 10 cases were Yunnan, Guizhou, and Sichuan Province (50, 33, 31 cases, respectively), accounting for 75% of all pediatric patients. In 2020, only one pediatric patient was discovered in the three above provinces, respectively. As for new case detection rate, Yunnan, Guizhou and Sichuan located in southwest China were also the top three provinces (Table 1).

**Table 1:** Spatial distribution of pediatric leprosy cases in China, 2011–2020.

Administrative region	Province	2011	2012	2013	2014	2015	2016	2017	2018	2019	2020	Total (NCDR per million)
**Northeast China**	Heilongjiang	0	0	0	0	0	0	0	0	0	0	0
	Jilin	0	0	0	0	0	0	0	0	0	0	0
	Liaoning	0	0	0	0	0	0	0	0	0	0	0
**North China**	Beijing	0	0	0	0	0	0	0	0	0	0	0
	Tianjin	0	0	0	0	0	0	0	0	0	0	0
	Hebei	0	0	0	0	0	0	0	0	0	0	0
	Shanxi	0	0	0	0	0	0	0	0	0	0	0
	Inner Mongolia	0	0	0	0	0	0	0	0	0	0	0
**East China**	Shanghai	0	0	0	0	0	0	0	0	0	0	0
	Jiangsu	0	0	0	0	0	0	0	0	0	0	0
	Zhejiang	0	1	0	0	0	0	0	0	0	0	1 (0.12)
	Anhui	1	0	0	0	1	0	0	0	0	0	2 (0.17)
	Fujian	0	1	1	1	0	0	0	0	0	0	3 (0.37)
	Jiangxi	1	0	0	1	0	1	1	1	0	0	5 (0.50)
	Shandong	0	0	0	0	0	0	0	0	0	0	0
**Central China**	Henan	0	0	1	0	1	0	0	0	0	0	2 (0.09)
	Hubei	0	0	0	0	0	1	0	0	0	0	1 (0.11)
	Hunan	1	0	0	0	1	0	0	0	0	0	2 (0.15)
**South China**	Guangdong	3	0	0	0	1	1	2	0	1	1	9 (0.38)
	Guangxi	2	1	0	0	2	1	0	0	2	1	9 (0.76)
	Hainan	0	0	0	0	0	0	0	0	0	0	0
**Southwest China**	Guizhou	5	14	4	3	0	3	2	1	0	1	33 (3.57)
	Yunnan	7	10	6	5	8	7	1	3	2	1	50 (5.41)
	Chongqing	0	1	0	0	0	0	0	0	0	0	1 (0.20)
	Sichuan	8	1	1	4	6	4	3	2	1	1	31 (2.30)
	Tibet	0	0	1	0	0	1	0	0	0	0	2 (2.28)
**Northwest China**	Shaanxi	0	0	0	0	0	0	0	0	0	0	0
	Gansu	1	0	0	0	0	0	0	0	0	0	1 (0.21)
	Qinghai	0	0	0	0	0	0	0	0	0	0	0
	Ningxia	0	0	0	0	0	0	0	0	0	0	0
	Xinjiang	0	0	0	0	0	0	0	0	0	0	0

NCDR = New Case Detection Rate

**Fig 2:**
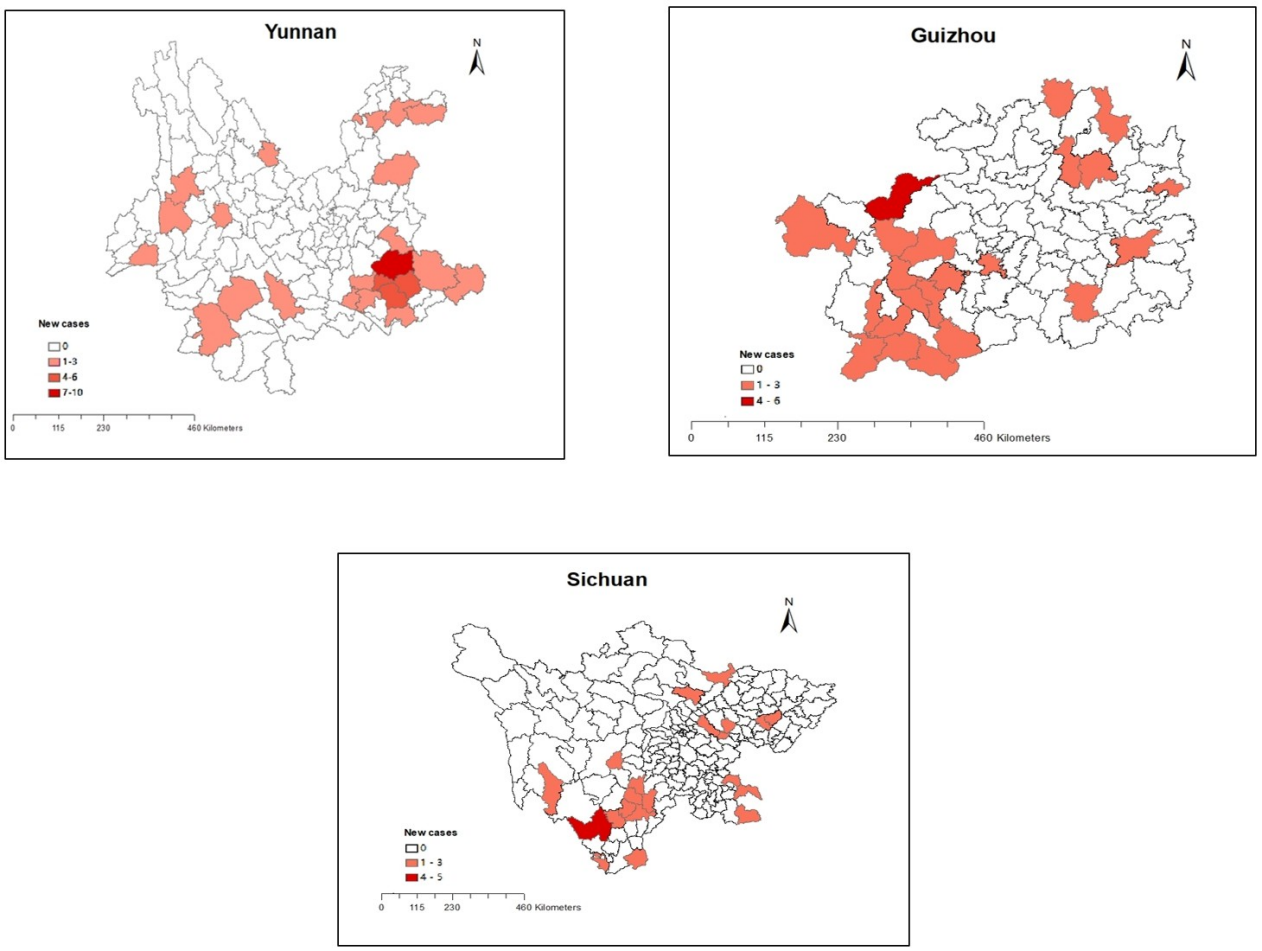
Spatial Distribution of pediatric new cases in Yunnan, Guizhou and Sichuan, China, 2011–2020. The base layers of the map are extracted from Naive Map developed in AMAP with data from the National Catalogue Service For Geographic Information. https://www.naivemap.com/admin-cn-downloader/.

The geographical distribution of new child leprosy cases in Yunnan, Guizhou and Sichuan is displayed in Fig 2. In Yunnan Province, 9 of 16 prefectures were detected new leprosy cases, among which Wenshan Zhuang and Miao Autonomous Prefecture in southeast Yunnan Province, Honghe Hani in southern part and Yi Autonomous Prefecture and Zhaotong City in northern part were the highest. At county level, the highest were found in Qiubei County, Wenshan City and Yanshan County located in Wenshan Zhuang and Miao Autonomous Prefecture. In Guizhou Province, Qianxinan Buyi and Miao Autonomous Prefecture, Bijie City, Anshun City and Tongren City had the highest number of new child leprosy cases. At county level, the highest one was Qixingguan District located in Bijie City. While in Sichuan Province, Liangshan Yi Autonomous Prefecture was the highest. At county level, Yanyuan County located in southwest region was reported the highest number of new child cases.

Among 152 new child cases, 96 were males and 56 were females, with a male-to-female ratio of 1.71:1. Over the last ten years, the average age of pediatric new cases was 10.8 years old. The youngest patient was 4 years old, while the oldest one was 14 years old. The majority of new child cases occurred in those aged 10 to 14 years old (74.3%). Meanwhile, a total of 123 new pediatric leprosy cases (80.9%) were HHCs (Table 2).

**Table 2:** Clinical and epidemiological characteristics of pediatric new cases according to WHO classification in China, 2011–2020.

Variables		WHO Classification		P-value
		MB (%)	PB (%)	
**Sex**	Male	87 (60.8)	9 (100)	0.027*
	Female	56 (39.2)	0	
**Age (years)**	0–4	1 (0.7)	1 (11.1)	0.021*
	5–9	33 (23.1)	4 (44.4)	
	10–14	109 (76.2)	4 (44.4)	
	**Diagnostic delay period(months)**	13.1±16.4	7.3±6.2	0.164**
^#^ **Number of lesions**	0	5 (3.6)	0	0.006*
	1	23 (16.5)	5 (62.5)	
	2–5	51 (36.7)	3 (37.5)	
	>5	60 (43.2)	0	
**Number of affected nerves**	0	41 (28.7)	9 (100)	<0.001*
	1	27 (18.9)	0	
	≥2	75 (52.4)	0	
^#^ **Physical disability degree**	Grade 0	127 (88.8)	9 (100)	>0.999*
	Grade 1	6 (4.2)	0	
	Grade 2	8 (5.6)	0	
**Leprosy reaction**	none	134 (93.7)	9 (100)	>0.999*
	Type I reaction	6 (4.2)	0	
	Type II reaction	3 (2.1)	0	
**Contact types**	HHCs	116 (81.1)	7 (77.8)	<0.681*
	Others	27 (18.9)	2 (22.2)	
**R-J Classification**	I	2 (1.4)	1 (11.1)	0.033*
	TT	25 (17.5)	4 (44.4)	
	BT	45 (31.5)	4 (44.4)	
	BB	15 (10.5)	0	
	BL	38 (26.6)	0	
	LL	18 (12.6)	0	

^#^There were missing values.

*Fisher’s exact test.

**Mann-Whitney test.

**Fig 3:**
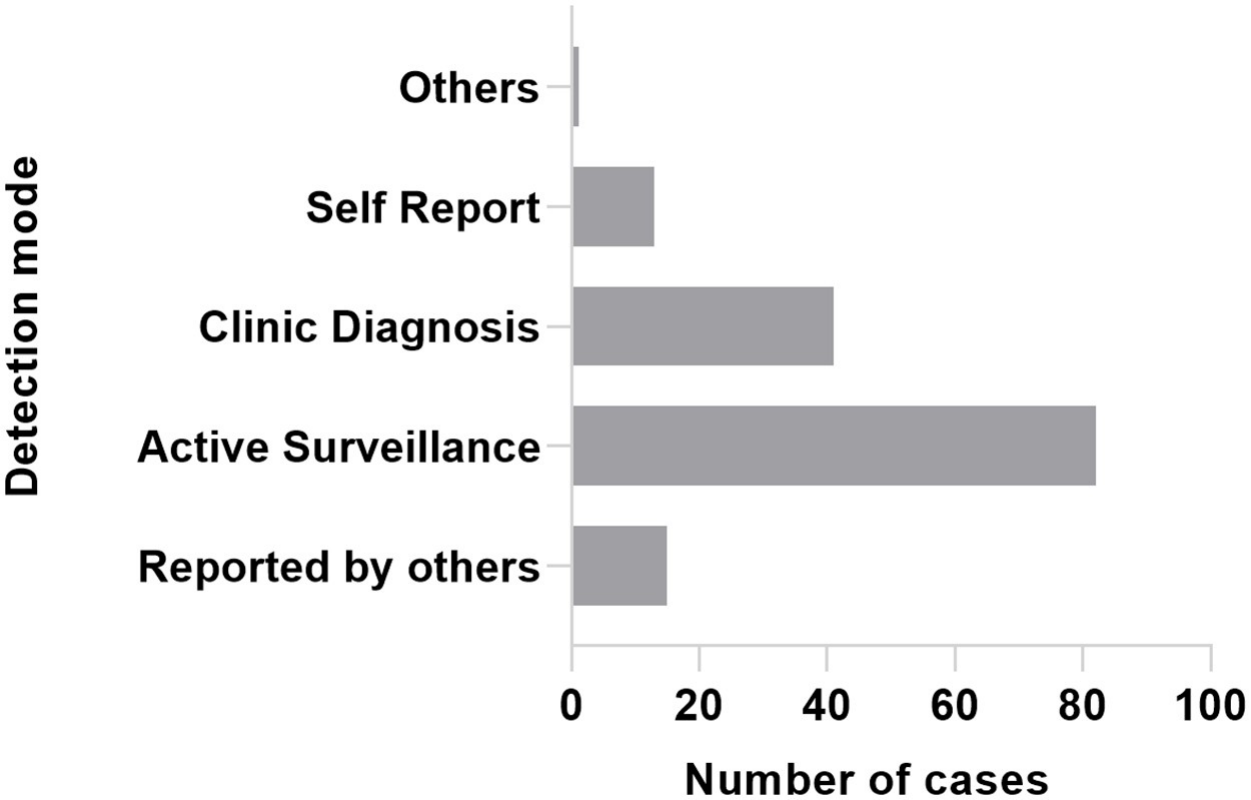
Detection modes of pediatric leprosy new cases in China, from 2011–2020.

Active surveillance included contact tracing, clue investigation, and community and endemic area surveys. As shown in Fig 3, 82 patients (53.9%) were detected through active surveillance, among which 59 cases (38.8%) were detected through contact tracing.

### Clinical characteristics

Among 152 pediatric new cases tested for the number of skin lesions, nearly 96% of patients developed skin lesions. A significant difference was found in the number of skin lesions between MB and PB patients (*P* = 0.006).

A total of 67.1% of pediatric leprosy new cases suffered nerve injury, among which more than 2 nerves were affected in 75 patients (49.3%). There was a significant difference in the number of affected nerves between MB and PB patients (*P*<0.001).

Only 9 patients (5.9%) were diagnosed with leprosy reactions. There were 6 patients with Type I reactions and 3 patients with Type II reactions. All of them were detected in MB patients.

Eight cases (5.3%) with G2D were detected. Surprisingly, pediatric patients with G2D are not included in the above 9 patients diagnosed with leprosy reactions. Although the MB group showed a higher rate of cases with G2D, no significant difference was found in cases with G2D between MB and PB cases (*P*>0.999).

## Discussion

Between 2010 and 2018, the new case detection rate of pediatric leprosy new cases presented a slight decreasing trend globally [12]. A total of 7472 newly detected leprosy cases were reported over the past decade in China. The new case detection rate has declined from 0.89 to 0.29 per million population from 2011 to 2020 in China. As is shown in our survey, 152 new pediatric cases were detected from 2011 to 2020 in China, indicating a reduction of 76.2% compared to the number of pediatric new cases from 1998 to 2010 (640) [20]. The new case detection rate of pediatric leprosy has decreased from 0.13 to 0.02 per million population over the past 10 years, which was much lower than that from 2001 to 2010. The downward trend indicates that our leprosy prevention and control strategies have made great progress in interrupting disease transmission and reducing the leprosy burden over the past 10 years. Despite this progress, the majority of sporadic cases still remain to be resolved. The leprosy new case detection rate in 2020 has not reached the goal of zero leprosy new child cases set by the WHO. There were still some sporadic new leprosy cases among children in the last three years. Hence, strategies for the prevention and control of leprosy should be continued.

We also observed that new leprosy cases among children mainly occurred in Yunnan, Guizhou, and Sichuan Provinces, in which there were 50, 33, and 31 cases, respectively. Similarly, the above three provinces were the top five high-burden areas in terms of overall new leprosy cases in China [10]. Yunnan, Guizhou, and Sichuan have always been classified as high-risk areas in China, suggesting clusters of leprosy infection in high-endemic areas in recent years. According to the definition given in a current WHO report [21], all but 7 provinces in China, including Sichuan, Yunnan, Guizhou, Guangxi, Tibet and Jiangxi, have achieved the goal of elimination of transmission. However, since new child cases in the above-mentioned provinces have fallen to zero or one case per year, they are approaching the cut-off line of ending transmission soon. At prefecture level, new child cases were clustered in Wenshan Zhuang and Miao Autonomous Prefecture in Yunnan Province, Qianxinan Buyi and Miao Autonomous Prefecture in Guizhou Province and Liangshan Yi Autonomous Prefecture in Sichuan Province. Short-range transmission and genetic susceptibility factors may play pivotal roles in occurrence of leprosy in the above high-endemic regions [22]. Eight-year molecular epidemiology study via multi-loci variable number of tandem repeats (VNTR) analysis in Quibei county of Wenshan Zhuang and Miao Autonomous Prefecture in Yunnan Province demonstrated that similar VNTR patterns were detected in patients from the same family and neighboring townships, which indicates intensive household contact and social contact through village markets [23,24]. The HLA-DRB1*13 allele was discovered to be related to leprosy in the Liangshan Yi Autonomous Region of Sichuan Province [25]. Although the majority of new child cases were detected in Yunnan, Guizhou and Sichuan, the number of child leprosy cases in these provinces presented a decreased trend over the past decade. This reduction is mainly attributed to the implementation of “Operational Guideline for Eliminating Harm due to Leprosy in China (2012–2020)” following the national leprosy control plan (2011 to 2020), which contributes to early detection, administration of MDT, professional training as well as health education for the public [26]. As for provinces without child leprosy cases, nearly all of them belong to low-endemic areas, which indicates that children in these provinces are less inclined to be exposed to leprosy patients, thus lowering the probability of infection.

New pediatric leprosy cases were more common in males, with a male to female ratio of 1.71:1. Similar results were discovered in a previous study in some provinces, such as Shaanxi in China [27]. The sex ratio results were also consistent with the findings in other countries or areas [15]. The higher proportion of males with leprosy may be attributed to sex differences in genetic susceptibilities [28]. However, a relatively higher proportion of females occurred in some poor areas [13,14]. This may be related to the unbalanced distribution of health care resources that results from sex discrimination. New leprosy cases among children tend to occur in individuals 10 to 14 years old (74.3%), which is consistent with the results reported in Brazil [15].

In our survey, 143 new patients were MB (76.3%). In contrast to our study, PB patients accounted for the majority of pediatric leprosy new cases in previous surveys [13,14,29]. MB leprosy patients are more likely to suffer severe clinical symptoms, leprosy reactions, and disabilities than PB leprosy patients. In our study, MB patients presented a higher number of skin lesions and affected nerves. This indicates the need to put great effort into screening strategies and early diagnosis so that we can detect infection early and lower the number of MB leprosy patients. The number of new cases with G2D reflects delayed diagnosis and its related factors, such as community awareness and the capacity of health care staff to recognize early symptoms and signs indirectly [12]. The risk factors associated with the presence of physical disability, including male sex, MB, leprosy reactions, and lepromatous type, were confirmed by a systematic review [30]. Although there was a higher proportion of male patients with MB, the proportion of pediatric patients with grade 2 disability accounted for less than 5% from 2011 to 2020. There were 9 new cases of leprosy reaction (5.9%). This finding indicates that we achieved a remarkable result in timely diagnosis and a high treatment completion rate.

Over 80% of pediatric leprosy cases were reported from HHCs in China, which is supported by previous work in 2016 in Salvador, Brazil [14,15]. Contacts of leprosy patients, especially HHCs, are susceptible to *M*. *leprae* infection. Research on predicting the risk of leprosy infection has identified various factors that contribute to the susceptibility of HHCs [16]. These factors might include gene susceptibility, being male and living with patients with MB. A prospective study confirmed that a nomogram integrating risk factors for developing leprosy could predict contacts at higher risk, stratify risk degree, and, thus, determine the target population for active surveillance [31]. More novel host biomarkers for point-of-care tests should be identified and applied to aid early diagnosis and large-scale contact screening [32].

Active surveillance consists of contact tracing, community tracing, clue surveys, and surveys in epidemic areas. We found that 53.9% of cases were detected through active surveillance. However, only 38.8% of leprosy cases were found through contact tracing. An active surveillance strategy plays a vital role in detecting new cases and tracing potential sources of infection compared to passive case finding [29]. Great effort should be made to improve contact tracing, including by strengthening the training of health care workers and enhancing the efficiency and accuracy of screening tests for leprosy.

There are still some limitations in this study. First, all epidemiological information was secondary data obtained from LEPMIS in China rather than directly collected by our researchers. However, LEPMIS was thought to be an authorized database used to notify leprosy cases that was updated regularly for over 10 years. LEPMIS is an essential database to upload the personal information of patients and their family members and report associated epidemiological and clinical features. This collection of data may contribute to active case surveillance and the timely detection of new patients. In addition, there were a few missing values in the degree of physical disability. Last but not least, according to the elimination standard of the WHO, China has become a low endemic country for leprosy since the last century. Epidemiological information and related control strategies may not be suitable for high-risk areas.

In conclusion, we observed a consistent decreasing tendency of new child cases of leprosy between 2011 and 2020 in China. New child cases were clustered in southwest and south of China. Leprosy transmission has ceased in the majority of provinces throughout China. But sporadic cases may still continue to appear for some time due to the very long incubation period. For high-risk areas, active surveillance should be strengthened in their counties with highest number of leprosy cases. Greater emphasis should be put on contact tracing, expanding the screening scope to near neighbors and regular examination annually. This relies on professional training and establishment of local facilities specialized in leprosy prevention and control. Early diagnosis and timely MDT therapy remain potent measures to control the infection effectively. For medium-risk and low-risk areas, the importation of leprosy cases from high-endemic areas should be strictly prevented while maintaining the existing management strategies.

## Supporting information

S1 Table Physical disability of pediatric new leprosy cases by leprosy reaction in China, 2011–2020. (DOCX)

S2 Table Diagnostic delay period of pediatric new leprosy cases by provinces in China, 2011–2020. (DOCX)

S3 Table Diagnostic delay period of pediatric new leprosy cases by endemic areas in China, 2011–2020. (DOCX)
