# Supplementary material for: Leprosy among new child cases in China: Epidemiological and clinical analysis from 2011 to 2020
Source: PLoS Negl Trop Dis. 2023 Feb 17;17(2):e0011092. doi: 10.1371/journal.pntd.0011092 (PMC9980728; doi:10.1371/journal.pntd.0011092)
Supplement: S1 Table — (DOCX) [file pntd.0011092.s001.docx]

**S4 Table. Physical disability of pediatric new leprosy cases by leprosy reaction in China, 2011-2020**

|  |  | **Leprosy reaction** | | |
| --- | --- | --- | --- | --- |
| **^#^Physical disability** |  | None | T1R | T2R |
|  | None | 128 | 6 | 2 |
|  | G1D | 5 | 0 | 1 |
|  | G2D | 8 | 0 | 0 |

^#^There were missing value. T1R=Type I Reaction. T2R=Type II Reaction.
