# Supplementary material for: Leprosy among new child cases in China: Epidemiological and clinical analysis from 2011 to 2020
Source: PLoS Negl Trop Dis. 2023 Feb 17;17(2):e0011092. doi: 10.1371/journal.pntd.0011092 (PMC9980728; doi:10.1371/journal.pntd.0011092)
Supplement: S2 Table — (DOCX) [file pntd.0011092.s002.docx]

**S5 Table. Diagnostic delay period of pediatric new leprosy cases by provinces in China, 2011-2020**

| **Province** | **Diagnostic delay period (months)** | **P-value** |
| --- | --- | --- |
| Anhui | 14.0±11.3 | 0.197 |
| Fujian | 3.0±3.6 |  |
| Gansu | 8.0 |  |
| Guangdong | 14.1±13.5 |  |
| Guangxi | 8.6±3.8 |  |
| Guizhou | 12.1±12.9 |  |
| Henan | 2.5±3.5 |  |
| Hubei | 44.0 |  |
| Hunan | 22.0±4.2 |  |
| Jiangxi | 13.4±14.1 |  |
| Sichuan | 14.7±19.9 |  |
| Tibet | 13.0±1.4 |  |
| Yunnan | 12.4±18.3 |  |
| Zhejiang | 7.0 |  |
| Chongqing | 27.0 |  |
