# Supplementary material for: Leprosy among new child cases in China: Epidemiological and clinical analysis from 2011 to 2020
Source: PLoS Negl Trop Dis. 2023 Feb 17;17(2):e0011092. doi: 10.1371/journal.pntd.0011092 (PMC9980728; doi:10.1371/journal.pntd.0011092)
Supplement: S3 Table — (DOCX) [file pntd.0011092.s003.docx]

**S6 Table. Diagnostic delay period of pediatric new leprosy cases by endemic areas in China, 2011-2020**

| **Endemic areas** | **Diagnostic delay period (months)** | **P-value** |
| --- | --- | --- |
| High-endemic area | 12.9±16.3 | 0.218 |
| Medium-endemic area | 13.1±13.6 |  |
| Low-endemic area | 2.5±3.5 |  |
